# Factors Associated with Changes in Retinal Layers Following Acute Optic Neuritis: A Longitudinal Study Using Optical Coherence Tomography

**DOI:** 10.3390/jcm9123857

**Published:** 2020-11-27

**Authors:** Yumi Lee, Kyung-Ah Park, Sei Yeul Oh, Ju-Hong Min, Byoung Joon Kim

**Affiliations:** 1 Department of Ophthalmology, Samsung Medical Center, Sungkyunkwan University School of Medicine, Seoul 06351, Korea; muyi709@Gmail.com (Y.L.); kparkoph@skku.edu (K.-A.P.); 2 Department of Ophthalmology, Chuncheon Sacred Heart Hospital, Hallym University Medical Center, Chuncheon 24253, Korea; 3 Department of Neurology, Samsung Medical Center, Sungkyunkwan University School of Medicine, Seoul 06351, Korea; bjkim@skku.edu

**Keywords:** acute optic neuritis, spectral-domain optical coherence tomography, retinal microstructures, longitudinal change, neuromyelitis optica

## Abstract

This study aimed to analyze longitudinal changes in retinal microstructures following acute optic neuritis and to identify the factors that affect those changes using spectral-domain optical coherence tomography (OCT). Forty-eight eyes of 37 patients with a first episode of optic neuritis and 48 eyes of 48 healthy controls were enrolled. Patients underwent serial OCT and visual function testing for more than six months. Individual layers from macular OCT were segmented with an automated algorithm. The total retinal layer (TRL), nerve fiber layer (NFL), ganglion cell layer (GCL) and inner plexiform layer (IPL) of optic neuritis eyes showed significant thinning with time over 6–15 months (*p* < 0.001 for all). The outer nuclear layer (ONL) showed a later decrease in thickness (*p* = 0.007). The outer retinal layer (ORL) showed an increase (*p* = 0.007) in thickness at two to five months which was sustained over time. Low visual acuity and neuromyelitis optica (NMO) immunoglobulin (Ig) G were associated with changes in the thickness of the GCL, IPL, and ONL over time (*p* < 0.05 for all). Low visual acuity was also associated with changes in the thickness of the NFL over time (*p* = 0.033). Dynamic changes of retinal microstructures varied according to the retinal layer examined after an optic neuritis attack. Initial visual acuity and NMO-IgG were found to be significant factors affecting the changes in thickness of each retinal layer. These results will lead to a better understanding of the pathologic changes that occur in eyes with optic neuritis.

## 1. Introduction

Optic neuritis (ON) is an acute demyelinating disease of the optic nerve with a variety of etiologies. In some patients it may be a symptom of neurologic conditions including multiple sclerosis (MS) [1], neuromyelitis optica spectrum disorder (NMOSD) [2], or other autoimmune diseases; however, for others it might represent isolated disease [3]. Although the prognosis of ON is good [4,5], most patients have lasting symptoms of visual impairment including subnormal visual acuity, visual field, color vision, and contrast sensitivity [6,7]. ON may induce permanent retrograde axonal injury. Changes in the thicknesses of the peripapillary retinal nerve fiber and retinal ganglion cell layers can indicate the severity of axonal damage after ON [8,9,10,11]. Several studies have reported that retinal layers other than ganglion cell layers are also affected in patients with ON, although the clinical significance of these changes is not clear [11,12]. To date, studies on alterations within each retinal layer over time in patients with ON are scarce [9,11,13]. We attempted to determine how initial visual acuity or the presence of NMO-IgG affects individual changes in macular thickness. Investigation of longitudinal changes in the retinal layer structures may help to understand the diverse clinical sequelae that occur following ON. Additionally, the results may be used to evaluate the efficacy of new therapeutic regimens. Thus, the objective of this study was to investigate and quantitate the temporal changes in retinal layers in patients with ON and to identify factors that significantly affect these retinal changes.

## 2. Experimental Section

We conducted this retrospective, longitudinal study on patients who displayed ON for the first time and visited Samsung Medical Center between April 2009 and September 2018. All cases were reviewed in accordance with the tenets of the Declaration of Helsinki and the study was approved by the Institutional Review Board of Samsung Medical Center. We confirmed each patient’s history of acute ON episodes through a review of medical charts. The diagnosis of an ON episode was based on clinical symptoms, such as gradual visual loss over several days with or without pain during eye movements, documented findings of decreased visual acuity, visual field defect, loss of color vision, relative afferent pupillary defect in case of unilateral involvement, and compatible fundus examination with or without signs of abnormal optic nerve enhancement on magnetic resonance imaging (MRI). We excluded patients with any of the following conditions: age less than 20 years or greater than 70 years, clinical features compatible with ischemic optic neuropathy, history of any form of neurological impairment, or diagnosis of a neurologic disorder such as NMOSD or MS prior to the first ON episode. We also excluded any patients with a history of systemic vasculitis, or a history or presence of malignancy or ocular pathology that could affect visual function including retinal disease and optic neuropathies other than ON such as glaucoma and compressive optic neuropathies. We also excluded patients with follow-up duration less than six months. The diagnosis of MS was based on the 2010 revised McDonald criteria [14] and NMOSD was diagnosed according to the international consensus diagnostic criteria [15]. We reviewed the following ophthalmologic data for all patients: initial visual acuity, color vision test score with Ishihara charts, slit-lamp biomicroscopy and fundus examination results, results of a visual field test with a Humphrey Field Analyzer following the 30-2 SITA-standard protocol, laboratory tests including test for neuromyelitis optica IgG (NMO-IgG), and clinical course.

As controls, we also recruited 48 disease-free subjects from staff and healthy volunteers, none of whom had a history of ocular or neurologic disease. We used data on only the right eye in the analysis except when the quality of the right eye data was insufficient for analyzing retinal layer thickness and the quality of the left eye data was suitable.

### 2.1. SD-OCT

**Figure 1:**
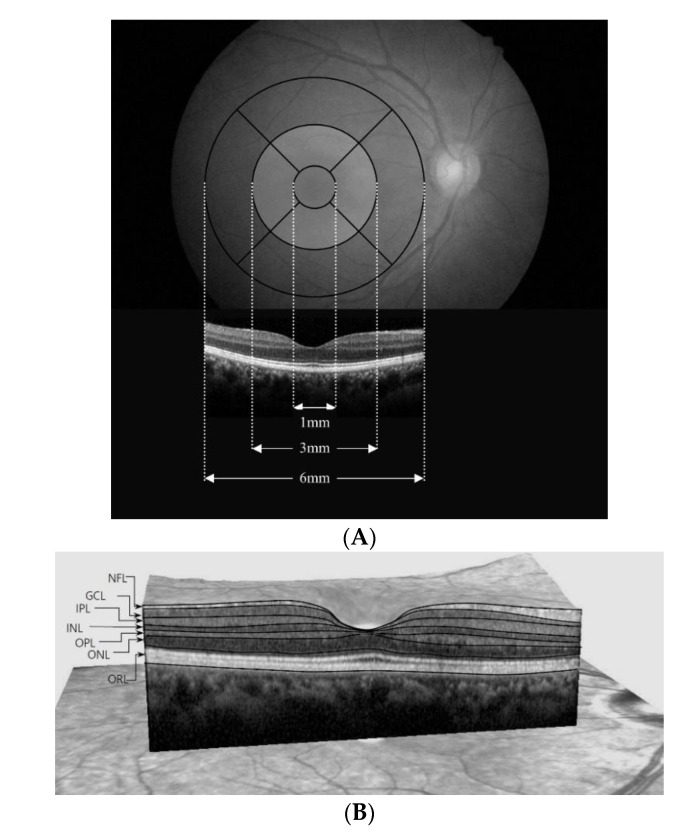
(**A**) The Early Treatment Diabetic Retinopathy Study area and spectral-domain optical coherence tomography (OCT) B-scans overlapped to the fundus. Foveal center thickness was defined as the mean value obtained at the foveal center from the horizontal and vertical scans. The thicknesses of inner locations, superior, inferior, nasal, and temporal, were the average measurements take at 490 μm and 500 μm in either direction from the foveal center. The thicknesses of the corresponding outer locations were defined as means measuring 1490 μm and 1500 μm in either direction from the foveal center. The thickness of each retinal layer was the sum of nine subfields. (**B**) Description of retinal segmentation of spectral-domain OCT. The segmented view of each retinal layer was labeled by the automated segmentation algorithm (with manual correction). NFL, Retinal nerve fiber layer; GCL, Ganglion cell layer; IPL, inner plexiform layer; INL, Inner nuclear layer; OPL, Outer plexiform layer; ONL, Outer nuclear layer; ORL, Outer retinal layer.

We used spectral-domain optical coherence tomography (SD-OCT) (Spectralis, Heidelberg Engineering, Heidelberg, Germany) to measure retinal thickness in all patients and healthy controls. Images were acquired using automated eye alignment eye-tracking software (TruTrack; Heidelberg Engineering) for improved image quality and segmentation accuracy, specifically, 25 single horizontal axial scans in the macular area. We only included high-quality scans (quality index > 20) in the study. We performed automated retinal segmentation using Spectralis software to identify individual retinal layers and quantify the thickness of each. We automatically measured the thickness of each layer between the outer limiting membrane and Bruch’s membrane and divided each into seven layers: the nerve fiber layer (NFL), ganglion cell layer (GCL), inner plexiform layer (IPL), outer plexiform layer (OPL), inner nuclear layer (INL), outer nuclear layer (ONL), and outer retinal layer (ORL), which ranged from the external limiting membrane to the basement membrane. The total retinal layer (TRL) was calculated as the sum of the thicknesses of all layers. For each layer, the SD-OCT calculated retinal thickness values of three concentric rings at 1 (central fovea), 3 (inner/parafoveal ring), and 6 mm (outer/perifoveal ring), dividing the inner and outer rings into four quadrants (nasal, superior, temporal, and inner) and creating nine total measurements per individual layer corresponding to Early Treatment Diabetic Retinopathy Study areas (Figure 1) [16], as well as the average thickness of each layer were used for statistical analysis. To assess the quality of the data, we used the Advised Protocol for OCT Study Terminology and Elements (APOSTEL) recommendations [17], which are based on validated OCT quality control criteria [18]. One clinician manually adjusted the segmentation results to ensure accuracy.

### 2.2. Statistical Analysis

We used Statistical Package for Social Sciences (SPSS) for Windows version 25 (IBM Corporation, Armonk, NY, USA) for all statistical analyses. Normality of distribution for continuous variables was confirmed by the Kolmogorov-Smirnov test. We used a linear regression model with generalized estimating equations (GEE) to compare between ON and control eyes at baseline and to analyze longitudinal changes in each retinal layer thickness in ON eyes after adjusting for age, gender, spherical equivalent refractive errors, and intra-subject inter-eye correlations. We also used GEE to analyze the interaction effects of covariates on thickening or thinning of each retinal layer: initial visual acuity and NMO-IgG. To investigate the effect of initial visual acuity when NMO-IgG was positive or negative, we conducted subgroup analysis by dividing the subjects into four groups: Group 1, NMO-IgG (−) and LogMAR < 1; Group 2, NMO-IgG (−) and LogMAR ≥ 1; Group 3, NMO-IgG (+) and LogMAR < 1; Group 4, NMO-IgG (+) and LogMAR ≥ 1. In the multivariate regression analysis, the comparison between Groups 1 and 2 revealed how initial visual acuity affected individual changes in macular thickness among patients positive for NMO-IgG, and the comparison between Groups 3 and 4 showed how initial visual acuity affected individual changes in macular thickness among patients negative for NMO-IgG. We report β coefficients with standard deviation for significant predictors, and we considered P values of less than 0.05 statistically significant. The association of changes in the OCT parameters with the final visual result was also analyzed using logistic regression models with GEE.

## 3. Results

### 3.1. Baseline Clinical Characteristics

Our retrospective study comprised 48 eyes of 37 patients with a first episode of ON and 48 eyes of 48 healthy controls. Demographic data on all participants are summarized in Table 1. The mean age of the ON patients was 39 ± 14 years (range, 20–66 years), and 13 (31%) were male; the mean follow-up duration was 11 ± 3 months (range, 6–15 months). The mean initial LogMAR visual acuity of ON patients was 1.20 ± 1.26 (range, 0.00–3.00). Of the 48 eyes with ON, 18 (38%) presented with an initial visual acuity of 20/200 or worse. The mean MD of the ON patients at the initial visual field examination was −15.88 ± 11.56 dB (range, −33.16–0.68 dB). Of the 48 eyes with ON, 28 (61%) had worse visual field function with MD values below −10 dB at the initial visit. Thirty (62%) patients showed disc swelling in the early phase. NMO-IgG was positive for 14 (39%) patients, and 3 (6%) patients were diagnosed with MS during the follow-up period. The mean LogMAR visual acuity of ON patients at the final visit was 0.72 ± 1.16 (range, 0.00–3.00). Of the 48 eyes with ON, 13 (27%) had final visual acuity of 20/200 or worse and 28 (58%) had final visual acuity of 20/25 or better. The mean MD of the ON patients at the final visual field examination was −9.31 ± 10.91 dB (range, −33.74–1.35 dB). Of the 48 eyes with ON, 16 (33%) had worse visual field function with MD values below −10 dB at the final visit.

### 3.2. Longitudinal Changes in Retinal Microstructures

After adjustment for intra-subject inter-eye correlations as well as age, gender, and refractive error, the TRL, GCL, and IPL of patients were significantly thinner at baseline than were those of controls (TRL *p* = 0.018; GCL *p* < 0.001; and IPL *p* = 0.002). However, the thicknesses of the other layers were not significantly different in patients vs. controls (Table 2). To identify the time point at which changes in thickness occurred, we divided the follow-up period into three time windows: less than one month after the onset of symptoms (Δ1), two to five months after onset (Δ2), and 6 to 15 months after onset (Δ3).

**Figure 2:**
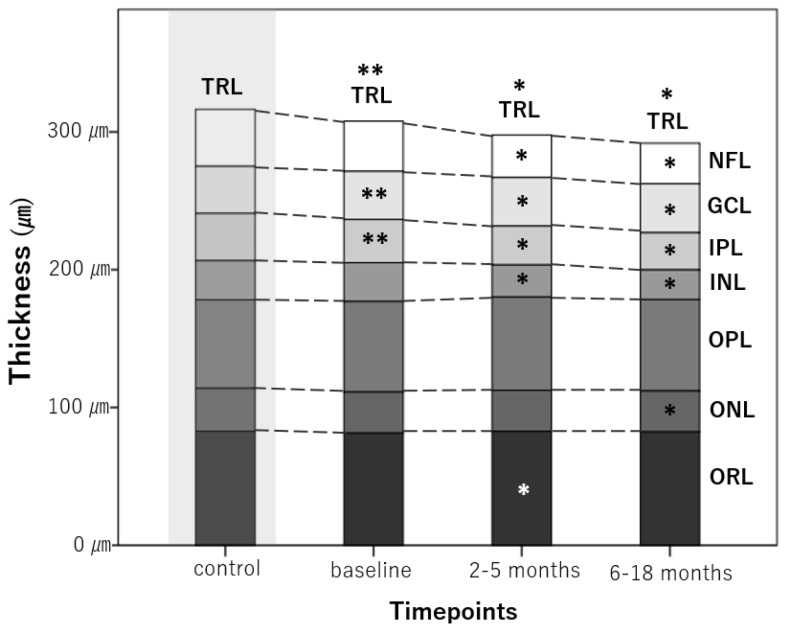
Longitudinal changes in average retinal layer thicknesses at different time points. A star (*) indicates a significant change in thickness compared with the previous time point. A double star (**) indicates a significant difference between control eyes and the first measurement taken in optic neuritis eyes. TRL, total macular volume; NFL, nerve fiber layer; GCL, ganglion cell layer; IPL, inner plexiform layer; INL, inner nuclear layer; OPL, outer plexiform layer; ONL, outer nuclear layer; ORL, outer retinal layer.

The TRL, NFL, GCL, and IPL of ON eyes showed significant longitudinal thinning throughout the whole period (*p* < 0.001 for all). The ONL showed a decrease in thickness at 6–15 months (*p* = 0.017). The thickness of the ORL increased at two to five months (*p* = 0.007). Figure 2 schematically illustrates the longitudinal changes in the thickness of each retinal layer.

### 3.3. Factors Associated with Changes in the Thickness of Each Retinal Layer

Table 3 and Table 4 show the factors that affected changes in the thickness of each retinal layer according to the linear regression model with GEE, with adjustment for intra-subject inter-eye correlations, gender, age, and refractive error.

Progressive thinning of the GCL and IPL over time was greater in patients positive for NMO-IgG compared to their negative counterparts (*p* = 0.017 and *p* = 0.031, respectively, not shown in the table), and those changes in thickness occur during the two to five months and 6–18 months following disease onset, respectively (GCL, Δ2-Δ1, β = −4.3, SD = 1.6, *p* = 0.028; IPL, Δ3-Δ1, β = −3.4, SD = 1.3, *p* = 0.025) (Table 3). The NFL was also thinner in patients positive for NMO-IgG (2–5 months, β = −3.1, SD = 1.2, *p* = 0.033) but the difference in thickness changes was not significant over time (*p* = 0.081, not shown in the table). The OPL was significantly thicker at 6–18 months in patients positive for NMO-IgG than in those negative for NMO-IgG (β = 2, SD = 0.8, *p* = 0.048), but the difference in thickness changes was not significant over time (*p* = 0.192, not shown in the table). ONL thickness reduced more in eyes positive for NMO-IgG than in those negative for NMO-IgG after 2-5 months. The difference in the thickness of the ONL according to the presence or absence of NMO-IgG was significantly reduced at 6–18 months compared to 2–5 months. (β = −2.1, SD = 0.8, *p* = 0.039).

Longitudinal thinning of the NFL, GCL and IPL was more severe in the eyes with low initial visual acuity (*p* = 0.033, *p* = 0.002, and *p* = 0.001, respectively, not shown in the table). The difference in thickness changes of NFL between low visual acuity group and better visual acuity group was significant during months 6–18 after disease onset (NFL, Δ3-Δ1, β = −3.5, SD = 1.4, p = 0.027), and the differences in changes in the GCL and IPL thicknesses were significant during months two to five after disease onset (GCL, Δ2-Δ1, β = −5.6, SD = 1.6, *p* = 0.001; IPL, Δ2-Δ1, β = −3.2, SD = 1.1, *p* = 0.012). ONL thickness in the group with low initial visual acuity showed less of a reduction at 6–18 months compared to the group with better visual acuity (β = 3.1, SD = 1.1, *p* = 0.013).

### 3.4. Subgroup Analysis

**Figure 3:**
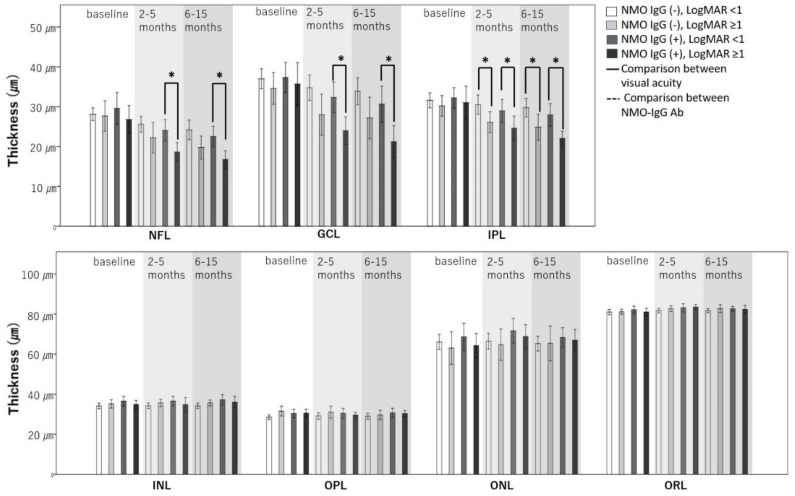
Changes in the thickness of each layer in the four groups over time. Interactions between time and group according to linear regression with GEE were significant at the *p* < 0.05 level; NFL *p* < 0.001; GCL *p* = 0.001; IPL *p* < 0.001; OPL *p* = 0.026; ONL *p* = 0.01. An asterisk (*) indicates a significant difference between the two groups.

Since both NMO-IgG and initial visual acuity significantly affected changes in retinal thickness, we divided all ON eyes into four groups to consider both factors simultaneously. The results of subgroup analysis are shown in Table 5 and Figure 3; other results are provided as Supplementary File 1 (Tables S1–S6).

Some layers of eyes with low initial visual acuity of LogMAR ≥ 1 showed a greater reduction in thickness than the same layers of eyes with relatively good initial visual acuity (LogMAR < 1). The decrease in thickness according to initial visual acuity differed depending on the presence or absence of NMO-IgG antibody. The difference in NFL thickness caused by low initial visual acuity was significant in eyes positive for NMO-IgG (2–5 months, β = −5.3, SD = 1.4, *p* = 0.001; 6–18 months, β = −5.6, SD = 1.3, *p* < 0.001), but not in eyes negative for NMO-IgG (2–5 months, β = −3.3, SD = 2,2, *p* = 0.383; 6–18 months, β = −4.4, SD = 2.0, *p* = 0.098). The degree of thickness reduction in GCL due to low initial visual acuity was greater in eyes positive for NMO-IgG (GCL, Δ3-Δ1, β = −7.8, SD = 3.1, *p* = 0.039) than eyes negative for NMO-IgG (GCL, Δ3-Δ1, β = −4.2, SD = 1.8, *p* = 0.053). In the IPL layer, however, a greater reduction in thickness was observed at low initial visual acuity regardless of the presence or absence of NMO-IgG Ab (NMO-IgG (+), Δ3-Δ1, β = −4.7, SD = 1.9, *p* = 0.048; NMO-IgG (−), Δ3-Δ1, β = −3.5, SD = 1.2, *p* = 0.013). In the NMO-IgG Ab-negative subgroup, the reduction in thickness according to initial visual acuity started earlier, from 2–5 months (NMO-IgG (−), Δ2-Δ1, β = −3.0, SD = 0.9, *p* = 0.003). In these same NMO-IgG-negative eyes, the difference in OPL thickness due to initial visual acuity showed a significant change over time (*p* = 0.026). The OPL exhibited a greater reduction in thickness in the subgroup with low initial visual acuity (Δ3-Δ1, β = −2.2, SD = 0.9, *p* = 0.034). All other layers showed no difference in thickness according to initial visual acuity when NMO-IgG Ab was positive or negative.

### 3.5. Associations between the Changes in Thickness of Each Retinal Layer over Time and Visual Field Outcomes

To determine the associations between the thickness of each retinal layer in each time period and visual field outcomes, we used logistic regression analyses with GEE for the macular retinal layers with adjustments for intra-subject inter-eye correlation, age, gender, and refractive errors. The patients were divided into two groups depending on the final visual field results. The patients in Group 1 had MD values greater than −10 dB, while those in Group 2 had MD values of −10 dB or less. The associations between the OCT parameters at each time point and worse final visual field outcomes (MD values of −10 dB or less) are shown in Table 6.

At baseline, within two months from disease onset, the OCT parameters that were significantly associated with worse final visual field outcomes were thinner nasal and inferior GCL (odds ratio (OR) = 0.87, *p* = 0.043, and OR = 0.84, *p* = 0.007) and inferior IPL (OR = 0.77, *p* = 0.01). At 2 to 5 months, the OCT parameters that were significantly associated with worse final visual field outcomes were thinner NFL, GCL and IPL in all sectors (all *p* < 0.05). At 6 to 18 months, thinner nasal and inferior TRL; thinner superior, nasal, and inferior NFL; thinner superior, inferior, and temporal GCL; and thinner inferior IPL were significantly associated with worse visual field outcomes (nasal TRL OR = 0.93, *p* = 0.047, inferior TRL OR = 0.92, *p* = 0.024, average NFL OR = 0.7, *p* = 0.022, superior NFL OR = 0.76, *p* = 0.007, nasal NFL OR = 0.78, *p* = 0.029, inferior NFL OR = 0.76, *p* = 0.039, average GCL OR = 0.76, *p* = 0.042, superior GCL OR = 0.81, *p* = 0.014, inferior GCL OR = 0.74, *p* = 0.024, temporal GCL OR = 0.79, *p* = 0.039, inferior IPL OR = 0.64, *p* = 0.016). There was no association between the thickness of the INL, OPL, ONL, or ORL at any time point and the visual field results.

## 4. Discussion

There were no significant differences in macular NFL thickness between control eyes and eyes with acute ON at baseline within 1 month of the onset of ON, while the GCL and IPL were thinner in eyes with acute ON than controls. However, the NFL thinned over time, as did the GCL and IPL, such that it was least thick at 6–15 months of follow-up. The differences in thickness of the NFL, GCL and IPL were greatest between one month and two to five months. There have been several previous reports on changes in the thickness of retinal layers over time using retinal segmentation analyses [9,13,19]. These studies concluded that the NFL, GCL, and IPL showed the most pronounced progressive thinning [9,13,19]. In agreement with those results, we also found that the thicknesses of the GCL and IPL in ON eyes were, on average, 15% and 10% lower, respectively, at two to five months and 19% and 14% lower at 6–15 months relative to baseline. The extent of thinning of the GCL and IPL in this study was slightly greater than that seen in the study by Al-Louzi et al., in which the ganglion cell and inner plexiform (GCIP) layer was thinner in affected eyes by an average of 11% at four months and 12% at 12 months [11]. This difference between studies might be attributable to differences in baseline characteristics such as age, ethnicity, and disease severity between the study populations.

In this study, initial visual acuity appeared to affect the gradual thickness reduction of the NFL, GCL, and IPL. Severe loss of vision initially may reflect increased disease severity, and initial vision is known to be one of the predictors of functional outcomes [20]. More remarkable changes in retinal layer thickness might reflect more severe axonal injury and disease severity in patients with severe initial visual loss than those who present with better initial vision.

Numerous investigators have found that NMOSD-ON eyes typically present more thinning over time in the RNFL and GCIPL than do MS-ON eyes [12,21,22,23,24]. In this study, the presence of NMO-IgG was associated with more pronounced thinning of the GCL and IPL. In NMO-IgG-positive eyes, the decrease in the thickness of those layers was more prominent in patients with low initial visual acuity.

Despite the potential overlap with other demyelinating disorders, NMOSD is known to lead to more severe astrocytic damage than demyelination, in contrast to other demyelinating diseases such as MS [25,26,27]. The important pathologic process in NMOSD is the production of abnormal autoantibodies against aquaporin-4 expressed on astrocytes [28]. These antibodies mediate complement-dependent astrocyte injury [29] along with neuronal loss [30]. Astrocytes are a type of glial cell that support and protect neurons in the central nervous system, including the retina [31]. The retinal ganglion cells (RGCs) are located in the GCL among the astrocytes; they send their axons toward the optic nerve and to the lateral geniculate nucleus and superior colliculus [32]. The processes of astrocytes form a honeycomb-shaped plexus that contributes to the GCL [33,34]. Astrocytes embryologically originate from the optic nerve [35], and astrocytes in the optic nerve support and nourish the axons of the RGCs [36,37]. NMOSD is more likely than other demyelinating diseases to lead to permanent visual deficits following attacks [38]. In our study, we confirmed that more GCL thinning occurred in NMO-IgG-positive than -negative patients, and GCL thinning was related to a prognosis of poor visual function. In addition, whether NMO-IgG was positive or negative, we found that low initial visual acuity was related to more severe GCL thinning. These findings may explain the high likelihood of permanent visual loss after an ON episode in patients with NMO with low initial visual acuity. The findings suggest that monitoring of retinal layer changes may be useful in the assessment of the efficacy of new therapeutic regimens in acute ON, especially ON associated with NMOSD.

In this study, ONL thickness showed a changing pattern over time: there was no difference in thickness according to NMO-IgG antibody positivity at each timepoints, but the thickness decreased faster after 2–5 months in eyes positive for NMO-IgG. In one previous study, ONL and photoreceptor segment thickness was high at four months and decreased reciprocally up to 12 months following acute ON [11]. Previous studies have reported thicker ONL in patients with NMOSD than patients with MS [11,12,19]. Two propositions have been suggested to explain ONL changes in patients with NMOSD. The first proposition is morphometric accommodation, in which other layers become thicker to compensate for the space created by the thinning of the ganglion cell layer [39]. The second relates to Müller cell dysfunction: Müller cell dysfunction may be promoted by trans-synaptic degeneration caused by ganglion cell layer thinning, which can lead to fluid accumulation and retinal thickening [13]. The inflammation in the acute phase of ON may also induce downregulation of Kir4.1 potassium channels present on Muller cells and, consequently, alter water homeostasis coupled with potassium transport [9]. The downregulation of Kir4.1 may lead to water accumulation and thickening of the outer layer [9].

This study has several limitations. First, we only used data from one ethnic group; thus, some results may not be valid in other ethnic groups. Additionally, the study was retrospective in design. Although the ON patients′ follow-up schedule was relatively uniform in our institute, there were some differences in follow-up intervals between a few patients. Several studies reported that macular edema varies over the course of a day in patients with diabetic retinopathy and central retinal vein occlusion [40,41,42]. The values obtained in the morning were higher than those in the afternoon or evening in the previous reports [40,41,42]. The variation was assumed to follow changes in blood pressure, retinal metabolism, and standing posture [40,42]. In another study investigating diurnal variations in macular thickness in healthy subjects, there were differences in diurnal variation according to the measurement device used [43]. In this study, in time domain (TD)-OCT, retinal thickness was significantly greater in the afternoon than in the morning among healthy participants [43]. In SD-OCT, however, there was no significant difference in macular thickness between the morning and the afternoon [43]. The authors explained that diurnal variation in the TD-OCT measurements was caused by a repeatability limitation of the device rather than by actual variation. In our study, SD-OCT with relatively good repeatability was used for measurement. However, there is a potential limitation associated with the inability to accurately repeat the OCT measurement over time.

Despite these deficiencies, we found significant longitudinal changes in retinal layers—the NFL, GCL, IPL, and ONL—following acute ON, and we also found significant associations between these changes and the factors of initial visual acuity and NMO-IgG. The thickness of the GCL and IPL was further reduced in patients positive for NMO-IgG. Additionally, in NMO-IgG-positive eyes that showed worse initial visual acuity, retinal layer thickness reduced to a greater extent than in eyes with better visual acuity. Our results will lead to a better understanding of the pathologic changes in the eye associated with ON. The clinical implications will need to be elucidated in greater detail through further long-term studies.

## Figures and Tables

**Table 1:** Demographics and clinical characteristics of patients with optic neuritis and healthy controls.

	Optic Neuritis (*n* = 48 Eyes)	Control (*n* = 48 Eyes)
Gender (Male/female) ^1^	13 (35%)/24 (65%)	23 (48%)/25 (52%)
Age ^2^	39 ± 14 (20,66)	40 ± 12 (23,64)
SE ^2^	−1.3 ± 2.0 (−5.8–1.13)	−0.9 ± 1.6 (−4.8–1.6)
Initial visual acuity (LogMAR)	1.20 ± 1.26 (0–3)	0 ± 0.02 (0–0.09)
Follow-up period (months)	11 ± 3 (6–15)	
NMO-IgG Ab (−/+)	28 (58%)/20 (42%)	
Disc swelling (−/+)	18 (38%)/30 (62%)	
Low visual acuity ^3^	18 (38%)	
Worse visual field ^4^	16 (33%)	

^1^ The gender distribution was compared using the chi-square test and found to be statistically insignificant (*p* = 0.668). ^2^ The Mann-Whitney U test was used to compare age and SE between two groups; the differences between groups were found to be statistically insignificant (*p* = 0. 350, *p* = 0.461, respectively). ^3^ Low visual acuity refers to an initial visual acuity of 20/200 or worse. ^4^ Worse visual field function is indicated by MD values of −10 dB or less at the final visit. SE: Spherical equivalent; LogMAR: logarithmic scale; NMO-IgG Ab: neuromyelitis optica-immunoglobulin G antibody.

**Table 2:** Mean retinal layer thickness of optic neuritis eyes and healthy control eyes over time.

	Control (*n* = 48 Eyes)	Optic Neuritis (*n* = 48 Eyes)			
		Time1 ^1^	Time2 ^2^	Time3 ^3^	
		Less than 1 Month	2–5 Months	6–15 Months	*p* Value
TRL (μm)	316.9 ± 12.5	307.8 ± 15.6	297.5 ± 15.5	291.4 ± 18.2	
*p* value		0.018	<0.001	<0.001	<0.001
NFL (μm)	28.5 ± 2.5	28.1 ± 4.5	23.3 ± 4.7	21.6 ± 5	
*p* value		1.000	<0.001	<0.001	<0.001
GCL (μm)	41.6 ± 2.8	36.4 ± 5.6	30.9 ± 7.2	29.5 ± 8	
*p* value		<0.001	<0.001	0.007	<0.001
IPL (μm)	75.9 ± 5	31.4 ± 4	28.2 ± 4.9	27 ± 5.1	
*p* value		0.002	<0.001	0.014	<0.001
INL (μm)	34 ± 2.8	35 ± 3	35.1 ± 3.4	35.5 ± 3.2	
*p* value		1.000	1.000	1.000	0.471
OPL (μm)	30.7 ± 4.2	29.9 ± 2.9	29.9 ± 3.2	29.8 ± 2.9	
*p* value		1.000	1.000	1.000	1.000
ONL (μm)	65.3 ± 6.9	65.9 ± 9	67.5 ± 8.7	66.3 ± 8.1	
*p* value		1.000	0.054	0.017	0.007
ORL (μm)	82.6 ± 2.3	81.3 ± 2.5	82.6 ± 2.3	82.2 ± 2.2	
*p* value		0.074	0.007	1.000	0.009

Generalized estimating equation (GEE) analysis for changes in retinal layer thickness over time with adjustments for intra-subject inter-eye correlations, age, gender, and refractive errors. ^1^ Linear regression model using generalized estimating equations (GEE) analysis between control and time 1. The corrected *p*-values adjusted with Bonferroni’s correction (multiplied by 8) for each outcome are shown. ^2^ Linear regression model using GEE analysis between time periods 1 and 2. The corrected *p*-values adjusted with Bonferroni’s correction (multiplied by 24) for each outcome are shown. ^3^ Linear regression model using GEE analysis between time periods 2 and 3. The corrected *p*-values adjusted with Bonferroni’s correction (multiplied by 24) for each outcome are shown. TRL: total retinal layer; NFL: nerve fiber layer; GCL: ganglion cell layer; IPL: inner plexiform layer; INL: inner nuclear layer; OPL: outer plexiform layer; ONL: outer nuclear layer; ORL: outer retinal layer.

**Table 3:** Effect of NMO-IgG on the change in thickness of each layer.

Retinal Layer	Difference in Thickness between MNO-IgG (+) vs. NMO IgG (−)			Difference in Thickness Changes between MNO-IgG (+) vs. NMO IgG (−)		
		β (SD)	*p*-Value		β (SD)	*p*-Value
NFL	Baseline	0 (1.3)	1.000	Δ2-Δ1	−3.1 (1.4)	0.088
	2–5 months	−3.1 (1.2)	0.033	Δ3-Δ2	0 (0.8)	1.000
	6–18 months	−3 (1.3)	0.068	Δ3-Δ1	−3.1 (1.4)	0.098
GCL ^1^	Baseline	−0.8 (1.8)	1.000	Δ2-Δ1	−4.3 (1.6)	0.028
	2–5 months	−5.1 (1.9)	0.022	Δ3-Δ2	−1.3 (0.8)	0.310
	6–18 months	−6.4 (2.1)	0.006	Δ3-Δ1	−5.6 (2)	0.013
IPL ^1^	Baseline	0.1 (1.4)	1.000	Δ2-Δ1	−2.6 (1.2)	0.074
	2–5 months	−2.5 (1.4)	0.228	Δ3-Δ2	−0.8 (0.7)	0.834
	6–18 months	−3.3 (1.5)	0.075	Δ3-Δ1	−3.4 (1.3)	0.025
INL	Baseline	1.6 (0.9)	0.219	Δ2-Δ1	−0.2 (0.7)	1.000
	2–5 months	1.4 (1.1)	0.563	Δ3-Δ2	0.9 (0.5)	0.175
	6–18 months	2.3 (1)	0.063	Δ3-Δ1	0.6 (0.5)	0.582
OPL	Baseline	1.6 (0.8)	0.114	Δ2-Δ1	−0.5 (0.6)	1.000
	2–5 months	1.1 (0.9)	0.722	Δ3-Δ2	0.9 (0.5)	0.210
	6–18 months	2 (0.8)	0.048	Δ3-Δ1	0.4 (0.6)	1.000
ONL ^1^	Baseline	−0.5 (2.5)	1.000	Δ2-Δ1	2.9 (1.3)	0.086
	2–5 months	2.4 (2.5)	0.983	Δ3-Δ2	−2.1 (0.8)	0.039
	6–18 months	0.3 (2.3)	1.000	Δ3-Δ1	0.8 (1.2)	1.000
ORL	Baseline	0.9 (0.8)	0.611	Δ2-Δ1	0.7 (0.7)	1.000
	2–5 months	1.6 (0.7)	0.067	Δ3-Δ2	−0.8 (0.6)	0.473
	6–18 months	0.8 (0.6)	0.672	Δ3-Δ1	−0.1 (0.7)	1.000

Generalized estimating equation (GEE) analysis of changes in retinal layer thickness over time with adjustments for intra-subject inter-eye correlations, age, gender, and refractive error. The corrected *p*-values adjusted with Bonferroni’s correction (multiplied by 3) for each outcome are shown. ^1^ Interactions between time and positive NMO-IgG Ab result according to GEE analysis that are significant at the *p* < 0.05 level; GCL *p* = 0.017; IPL *p* = 0.031; ONL *p* = 0.023. Δ1 refers to the difference in thickness between NMO-IgG (+) and NMO-IgG (−) eyes at baseline. Δ2 refers to the difference in thickness between NMO-IgG (+) and NMO-IgG (−) eyes at 2–5 months after onset of optic neuritis. Δ3 refers to the difference in thickness between NMO-IgG (+) and NMO-IgG (−) eyes at 6–15 months after onset of optic neuritis. NMO-IgG: neuromyelitis optica-immunoglobulin G; NFL: nerve fiber layer; GCL: ganglion cell layer; IPL: inner plexiform layer; INL: inner nuclear layer; OPL: outer plexiform layer; ONL: outer nuclear layer; ORL: outer retinal layer.

**Table 4:** Effect of initial visual acuity on the change in thickness of each layer.

Retinal Layer	Difference in Thickness between LogMAR ≥ 1 vs. LogMAR < 1			Difference in Thickness Changes between LogMAR ≥ 1 vs. LogMAR < 1		
		β (SD)	*p*-Value		β (SD)	*p*-Value
NFL ^1^	Baseline	−1.4 (1.2)	0.748	Δ2-Δ1	−2.9 (1.4)	0.11
	2–5 months	−4.3 (1.3)	0.003	Δ3-Δ2	−0.7 (0.8)	1.000
	6–18 months	−4.9 (1.3)	< 0.001	Δ3-Δ1	−3.5 (1.4)	0.027
GCL ^1^	Baseline	−1.4 (1.8)	1.000	Δ2-Δ1	−5.6 (1.6)	0.001
	2–5 months	−7 (2)	0.001	Δ3-Δ2	−0.7 (0.9)	1.000
	6–18 months	−7.7 (2.2)	0.001	Δ3-Δ1	−6.3 (1.9)	0.003
IPL ^1^	Baseline	−1.1 (1.2)	1.000	Δ2-Δ1	−3.2 (1.1)	0.012
	2–5 months	−4.3 (1.3)	0.003	Δ3-Δ2	−1.1 (0.7)	0.373
	6–18 months	−5.4 (1.2)	0.001	Δ3-Δ1	−4.3 (1.2)	0.001
INL	Baseline	−0.2 (0.8)	1.000	Δ2-Δ1	0.1 (0.7)	1.000
	2–5 months	0 (1)	1.000	Δ3-Δ2	0.4 (0.5)	1.000
	6–18 months	0.4 (0.9)	1.000	Δ3-Δ1	0.6 (0.5)	0.914
OPL	Baseline	1.5 (0.8)	0.233	Δ2-Δ1	−1.1 (0.6)	0.231
	2–5 months	0.4 (0.9)	1.000	Δ3-Δ2	−0.3 (0.5)	1.000
	6–18 months	0.1 (0.8)	1.000	Δ3-Δ1	−1.4 (0.6)	0.068
ONL ^1^	Baseline	−1.5 (2.7)	1.000	Δ2-Δ1	1.7 (1.3)	0.627
	2–5 months	0.2 (2.7)	1.000	Δ3-Δ2	1.4 (0.9)	0.294
	6–18 months	1.6 (2.6)	1.000	Δ3-Δ1	3.1 (1.1)	0.013
ORL	Baseline	−0.5 (0.7)	1.000	Δ2-Δ1	1.1 (0.7)	0.334
	2–5 months	0.6 (0.6)	0.972	Δ3-Δ2	−0.4 (0.5)	1.000
	6–18 months	0.3 (0.7)	1.000	Δ3-Δ1	0.7 (0.7)	0.869

Generalized estimating equation (GEE) analysis of changes in retinal layer thickness over time with adjustments for intra-subject inter-eye correlations, age, gender, and refractive errors. The corrected *p*-values adjusted with Bonferroni’s correction (multiplied by 3) for each outcome are shown. ^1^ Interactions between time and visual acuity according to GEE analysis that are significant at the *p* < 0.05 level; NFL *p* = 0.033; GCL *p* = 0.002; IPL *p* = 0.001; ONL *p* = 0.005. Δ1 refers to the difference in thickness depending on visual acuity at baseline. Δ2 refers to the difference in thickness depending on visual acuity at 2–5 months after onset of optic neuritis. Δ3 refers to the difference in thickness depending on visual acuity at 6–15 months after onset of optic neuritis. LogMAR: Logarithmic scale; NFL: nerve fiber layer; GCL: ganglion cell layer; IPL: inner plexiform layer; INL: inner nuclear layer; OPL: outer plexiform layer; ONL: outer nuclear layer; ORL: outer retinal layer.

**Table 5:** Subgroup analysis showing differences in the effect of low visual acuity on changes in thickness between groups.

		Difference in Thickness between LogMAR ≥ 1 vs. LogMAR < 1			
		In Eyes without NMO-IgG		In Eyes with NMO-IgG	
		β	*p*-Value	β	*p*-Value
NFL ^1^	Δ1	−0.5 (1.9)	1.000	−2.6 (1.9)	0.493
	Δ2-Δ1	−2.8 (1.8)	0.364	−2.6 (2.3)	0.765
	Δ3-Δ2	−1 (0.9)	0.807	−0.3 (1.4)	1.000
	Δ3-Δ1	−3.9 (1.7)	0.075	−2.9 (2.3)	0.582
GCL ^1^	Δ1	−2.1 (2.6)	1.000	−1.5 (2.6)	1.000
	Δ2-Δ1	−4.3 (1.5)	0.013	−6.7 (2.6)	0.031
	Δ3-Δ2	0.1 (1)	1.000	−1.1 (1.4)	1.000
	Δ3-Δ1	−4.2 (1.8)	0.053	−7.8 (3.1)	0.039
IPL ^1^	Δ1	−2.2 (1.6)	0.504	−1.2 (2)	1.000
	Δ2-Δ1	−3 (0.9)	0.003	−3.1 (2)	0.363
	Δ3-Δ2	−0.5 (1)	1.000	−1.6 (1)	0.369
	Δ3-Δ1	−3.5 (1.2)	0.013	−4.7 (1.9)	0.048
INL	Δ1	0.1 (1)	1.000	−1.9 (1.3)	0.419
	Δ2-Δ1	0.4 (0.5)	1.000	−0.1 (1.4)	1.000
	Δ3-Δ2	0 (0.5)	1.000	0.6 (0.9)	1.000
	Δ3-Δ1	0.4 (0.7)	1.000	0.5 (0.9)	1.000
OPL ^1^	Δ1	2.4 (1.2)	0.148	0 (1.2)	1.000
	Δ2-Δ1	−1 (1)	0.915	−1.1 (0.8)	0.483
	Δ3-Δ2	−1.2 (0.7)	0.31	0.6 (0.7)	1.000
	Δ3-Δ1	−2.2 (0.9)	0.034	−0.5 (0.8)	1.000
ONL ^1^	Δ1	−0.3 (4.1)	1.000	−3.6 (3.7)	0.993
	Δ2-Δ1	1.3 (1.8)	1.000	1.6 (2.1)	1.000
	Δ3-Δ2	1.8 (1.2)	0.378	1.5 (1.2)	0.681
	Δ3-Δ1	3.1 (1.4)	0.079	3 (1.8)	0.288
ORL	Δ1	−0.4 (0.7)	1.000	−1.6 (1)	0.339
	Δ2-Δ1	0.8 (0.9)	1.000	1.5 (1.1)	0.528
	Δ3-Δ2	0.1 (0.7)	1.000	−0.6 (0.9)	1.000
	Δ3-Δ1	0.9 (0.8)	0.846	0.9 (1.2)	1.000

Generalized estimating equation (GEE) analysis of changes in retinal layer thickness over time with adjustments for intra-subject inter-eye correlations, age, gender, and refractive error. The corrected *p*-values adjusted with Bonferroni’s correction (multiplied by 3) for each outcome are shown. ^1^ Interactions between time and visual acuity according to GEE analysis that are significant at the *p* < 0.05 level; NFL *p* < 0.001; GCL *p* = 0.001; IPL *p* < 0.001; OPL *p* = 0.026; ONL *p* = 0.01. Δ1 refers to the difference in thickness between two groups. Δ2 refers to the difference in thickness between two groups at 2–5 months after onset of optic neuritis. Δ3 refers to the difference in thickness between two groups at 6–15 months after onset of optic neuritis. LogMAR: Logarithmic scale; NMO-IgG: neuromyelitis optica-immunoglobulin G; NFL: nerve fiber layer; GCL: ganglion cell layer; IPL: inner plexiform layer; INL: inner nuclear layer; OPL: outer plexiform layer; ONL: outer nuclear layer; ORL: outer retinal layer.

**Table 6:** Association between OCT parameters at each time point and worse visual field outcomes (MD values of −10 dB or less) in ON eyes.

Retinal Layer		Baseline		2–5 Months		6–18 Months	
		OR (95% CI)	*p*-Value	OR (95% CI)	*p*-Value	OR (95% CI)	*p*-Value
TRL	Average	0.96 (0.91, 1.01)	0.395	0.92 (0.84, 1)	0.168	0.92 (0.85, 0.99)	0.077
	Superior	0.97 (0.92, 1.02)	0.606	0.93 (0.87, 1)	0.114	0.93 (0.87, 0.99)	0.051
	Nasal	0.97 (0.94, 1.01)	0.388	0.94 (0.88, 1)	0.112	0.93 (0.88, 0.99)	0.047
	Inferior	0.96 (0.91, 1.01)	0.232	0.92 (0.86, 0.99)	0.057	0.92 (0.86, 0.98)	0.024
	Temporal	0.96 (0.9, 1.01)	0.414	0.93 (0.86, 1)	0.115	0.93 (0.87, 0.99)	0.073
NFL	Average	0.88 (0.73, 1.06)	0.506	0.78 (0.65, 0.93)	0.019	0.7 (0.54, 0.91)	0.022
	Superior	0.93 (0.82, 1.05)	0.659	0.84 (0.75, 0.95)	0.013	0.76 (0.64, 0.91)	0.007
	Nasal	0.96 (0.87, 1.07)	1.000	0.85 (0.76, 0.96)	0.027	0.78 (0.64, 0.94)	0.029
	Inferior	0.88 (0.78, 0.99)	0.107	0.82 (0.71, 0.96)	0.036	0.76 (0.61, 0.94)	0.039
	Temporal	0.61 (0.37, 0.99)	0.141	0.61 (0.41, 0.9)	0.037	0.6 (0.36, 0.97)	0.116
GCL	Average	0.85 (0.75, 0.97)	0.054	0.79 (0.66, 0.94)	0.023	0.76 (0.6, 0.95)	0.042
	Superior	0.9 (0.81, 1)	0.122	0.85 (0.76, 0.96)	0.022	0.81 (0.7, 0.94)	0.014
	Nasal	0.87 (0.78, 0.97)	0.043	0.8 (0.69, 0.94)	0.016	0.8 (0.66, 0.96)	0.054
	Inferior	0.84 (0.74, 0.94)	0.007	0.77 (0.64, 0.93)	0.018	0.74 (0.59, 0.92)	0.024
	Temporal	0.89 (0.79, 1.01)	0.193	0.83 (0.71, 0.96)	0.045	0.79 (0.66, 0.95)	0.039
IPL	Average	0.79 (0.65, 0.97)	0.066	0.69 (0.52, 0.91)	0.024	0.67 (0.48, 0.93)	0.054
	Superior	0.82 (0.69, 0.99)	0.111	0.69 (0.53, 0.9)	0.019	0.71 (0.53, 0.95)	0.059
	Nasal	0.84 (0.72, 0.98)	0.081	0.75 (0.59, 0.94)	0.040	0.71 (0.53, 0.95)	0.064
	Inferior	0.77 (0.64, 0.92)	0.010	0.66 (0.5, 0.88)	0.013	0.64 (0.47, 0.88)	0.016
	Temporal	0.8 (0.66, 0.99)	0.106	0.74 (0.58, 0.94)	0.037	0.71 (0.53, 0.94)	0.056
INL	Average	1.04 (0.8, 1.35)	1.000	1.14 (0.91, 1.43)	0.738	1.15 (0.93, 1.43)	0.624
	Superior	1.07 (0.89, 1.28)	1.000	1.13 (0.98, 1.32)	0.299	1.14 (0.98, 1.31)	0.256
	Nasal	0.99 (0.81, 1.21)	1.000	1.11 (0.95, 1.3)	0.612	1.12 (0.97, 1.3)	0.407
	Inferior	1.1 (0.86, 1.4)	1.000	1.11 (0.88, 1.39)	1.000	1.07 (0.85, 1.35)	1.000
	Temporal	0.99 (0.77, 1.28)	1.000	1.06 (0.84, 1.34)	1.000	1.08 (0.87, 1.34)	1.000
OPL	Average	1.04 (0.87, 1.25)	1.000	0.96 (0.82, 1.13)	1.000	1.03 (0.86, 1.23)	1.000
	Superior	1.05 (0.95, 1.16)	0.978	0.98 (0.9, 1.07)	1.000	1.07 (0.95, 1.2)	0.867
	Nasal	0.98 (0.86, 1.12)	1.000	0.94 (0.83, 1.06)	0.882	0.94 (0.83, 1.07)	1.000
	Inferior	1.03 (0.89, 1.2)	1.000	1.07 (0.96, 1.2)	0.678	1.03 (0.93, 1.15)	1.000
	Temporal	1.07 (0.91, 1.25)	1.000	1.01 (0.86, 1.18)	1.000	1.13 (0.91, 1.39)	0.843
ONL	Average	1.02 (0.95, 1.09)	1.000	1.04 (0.98, 1.12)	0.646	1.05 (0.98, 1.13)	0.493
	Superior	1.01 (0.95, 1.07)	1.000	1.04 (0.98, 1.11)	0.569	1.02 (0.96, 1.09)	1.000
	Nasal	1.03 (0.97, 1.09)	1.000	1.05 (0.98, 1.12)	0.446	1.06 (0.99, 1.14)	0.243
	Inferior	1.01 (0.95, 1.07)	1.000	1.01 (0.95, 1.07)	1.000	1.03 (0.96, 1.11)	1.000
	Temporal	1.01 (0.95, 1.08)	1.000	1.03 (0.97, 1.09)	1.000	1.04 (0.97, 1.11)	0.717
ORL	Average	0.85 (0.61, 1.18)	1.000	1.05 (0.8, 1.38)	1.000	0.97 (0.72, 1.29)	1.000
	Superior	0.82 (0.64, 1.05)	0.348	0.95 (0.73, 1.22)	1.000	0.99 (0.76, 1.28)	1.000
	Nasal	0.8 (0.57, 1.11)	0.555	1.02 (0.83, 1.26)	1.000	0.95 (0.73, 1.24)	1.000
	Inferior	0.93 (0.7, 1.23)	1.000	1.06 (0.84, 1.33)	1.000	1 (0.78, 1.29)	1.000
	Temporal	0.88 (0.68, 1.14)	0.960	1.09 (0.82, 1.46)	1.000	1.04 (0.77, 1.4)	1.000

Logistic regression analysis using the generalized estimating equation for the association between retinal layer thickness and visual field outcome with adjustments for intra-subject inter-eye correlations, age, gender, and refractive error. The corrected *p*-values adjusted with Bonferroni’s correction (multiplied by 3) for each outcome are shown. CI: confidence interval; OR: odds ratio; TRL: total retinal layer; NFL: nerve fiber layer; GCL: ganglion cell layer; IPL: inner plexiform layer; INL: inner nuclear layer; OPL: outer plexiform layer; ONL: outer nuclear layer; ORL: outer retinal layer.

## Supplementary Materials

The following are available online at https://www.mdpi.com/2077-0383/9/12/3857/s1, Table S1: Changes in the thickness of each retinal layer between Groups 1 and 2, Table S2: Changes in the thickness of each retinal layer between Groups 3 and 4, Table S3: Changes in the thickness of each retinal layer between Groups 1 and 3, Table S4: Changes in the thickness of each retinal layer between Groups 2 and 4, Table S5: Changes in the thickness of each retinal layer between Groups 1 and 4, Table S6: Changes in the thickness of each retinal layer between Groups 2 and 3.
