# Supplementary material for: Factors Associated with Changes in Retinal Layers Following Acute Optic Neuritis: A Longitudinal Study Using Optical Coherence Tomography
Source: J Clin Med. 2020 Nov 27;9(12):3857. doi: 10.3390/jcm9123857 (PMC7760276; doi:10.3390/jcm9123857)
Supplement: Supplementary file 1 [file jcm-09-03857-s001.pdf]

Journal of
Clinical Medicine

MDPI

Supplementary Materials

For the subanalysis considering the low initial visual acuity and NMO-IgG, it was divided into 4 groups as follows; Group 1, NMO-IgG (-) and LogMAR < 1; Group 2, NMO-IgG (-) and LogMAR ≥ 1; Group 3, NMO-IgG (+) and LogMAR < 1; Group 4, NMO-IgG (+) and LogMAR ≥ 1. The six tables show the results of the linear regression analysis using GEE between the two groups.

Table S1. Changes of thickness in each retinal layer between group 1 and 2.

Retinal layer	Difference in thickness Between NMO-IgG (−) and LogMAR < 1 vs. NMO-IgG (−) and LogMAR ≥ 1			Changes in thickness difference between NMO-IgG (−) and LogMAR < 1 vs. NMO-IgG (−) and LogMAR ≥ 1		
		β	*p* value		β	*p* value
NFL	Baseline	−0.5(1.9)	1.000	Δ2–Δ1	−2.8(1.8)	0.364
	2–5 months	−3.3(2.2)	0.383	Δ3–Δ2	−1(0.9)	0.807
	6–18 months	−4.4(2)	0.098	Δ3–Δ1	−3.9(1.7)	0.075
GCL	Baseline	−2.1(2.6)	1.000	Δ2–Δ1	−4.3(1.5)	**0.013**
	2–5 months	−6.4(3.1)	0.114	Δ3–Δ2	0.1(1)	1.000
	6–18 months	−6.3(3.2)	0.144	Δ3–Δ1	−4.2(1.8)	0.053
IPL	Baseline	−2.2(1.6)	0.504	Δ2–Δ1	−3(0.9)	**0.003**
	2–5 months	−5.2(1.8)	**0.012**	Δ3–Δ2	−0.5(1)	1.000
	6–18 months	−5.7(1.8)	**0.006**	Δ3–Δ1	−3.5(1.2)	**0.013**
INL	Baseline	0.1(1)	1.000	Δ2–Δ1	0.4(0.5)	1.000
	2–5 months	0.5(0.9)	1.000	Δ3–Δ2	0(0.5)	1.000
	6–18 months	0.5(0.9)	1.000	Δ3–Δ1	0.4(0.7)	1.000
OPL	Baseline	2.4(1.2)	0.148	Δ2–Δ1	−1(1)	0.915
	2–5 months	1.3(1.5)	1.000	Δ3–Δ2	−1.2(0.7)	0.31
	6–18 months	0.1(1.2)	1.000	Δ3–Δ1	−2.2(0.9)	**0.034**
ONL	Baseline	−0.3(4.1)	1.000	Δ2–Δ1	1.3(1.8)	1.000
	2–5 months	1(4.3)	1.000	Δ3–Δ2	1.8(1.2)	0.378
	6–18 months	2.8(4.3)	1.000	Δ3–Δ1	3.1(1.4)	0.079
ORL	Baseline	−0.4(0.7)	1.000	Δ2–Δ1	0.8(0.9)	1.000
	2–5 months	0.4(1)	1.000	Δ3–Δ2	0.1(0.7)	1.000
	6–18 months	0.5(1)	1.000	Δ3–Δ1	0.9(0.8)	0.846

Table S2. Changes of thickness in each retinal layer between group 3 and 4.

Retinal layer	Difference in thickness Between NMO-IgG (+) and LogMAR ≥ 1 vs. NMO-IgG (+) and LogMAR < 1			Changes in thickness difference between NMO-IgG (+) and LogMAR ≥ 1 vs. NMO-IgG (+) and LogMAR < 1		
		β	*p* value		β	*p* value
NFL	Baseline	−2.6(1.9)	0.493	Δ2–Δ1	−2.6(2.3)	0.765
	2–5 months	−5.3(1.4)	**0.001**	Δ3–Δ2	−0.3(1.4)	1.000
	6–18 months	−5.6(1.3)	**<0.001**	Δ3–Δ1	−2.9(2.3)	0.582
GCL	Baseline	−1.5(2.6)	1.000	Δ2–Δ1	−6.7(2.6)	**0.031**
	2–5 months	−8.2(2.1)	**<0.001**	Δ3–Δ2	−1.1(1.4)	1.000
	6–18 months	−9.3(2.6)	**0.001**	Δ3–Δ1	−7.8(3.1)	**0.039**
IPL	Baseline	−1.2(2)	1.000	Δ2–Δ1	−3.1(2)	0.363
	2–5 months	−4.3(1.8)	**0.041**	Δ3–Δ2	−1.6(1)	0.369
	6–18 months	−5.9(1.4)	**<0.001**	Δ3–Δ1	−4.7(1.9)	**0.048**
INL	Baseline	−1.9(1.3)	0.419	Δ2–Δ1	−0.1(1.4)	1.000

	2–5 months	−2(1.8)	0.738	Δ3–Δ2	0.6(0.9)	1.000
	6–18 months	−1.4(1.6)	1.000	Δ3–Δ1	0.5(0.9)	1.000
OPL	Baseline	0(1.2)	1.000	Δ2–Δ1	−1.1(0.8)	0.483
	2–5 months	−1.1(1.2)	1.000	Δ3–Δ2	0.6(0.7)	1.000
	6–18 months	−0.5(1.2)	1.000	Δ3–Δ1	−0.5(0.8)	1.000
ONL	Baseline	−3.6(3.7)	0.993	Δ2–Δ1	1.6(2.1)	1.000
	2–5 months	−2(3.5)	1.000	Δ3–Δ2	1.5(1.2)	0.681
	6–18 months	−0.5(3)	1.000	Δ3–Δ1	3(1.8)	0.288
ORL	Baseline	−1.6(1)	0.339	Δ2–Δ1	1.5(1.1)	0.528
	2–5 months	−0.1(1)	1.000	Δ3–Δ2	−0.6(0.9)	1.000
	6–18 months	−0.7(0.9)	1.000	Δ3–Δ1	0.9(1.2)	1.000

Table S3. Changes of thickness in each retinal layer between group 1 and 3.

Retinal layer	Difference in thickness Between NMO-IgG (+) and LogMAR < 1 vs. NMO-IgG (−) and LogMAR < 1			Changes in thickness difference between NMO-IgG (+) and LogMAR < 1 vs. NMO-IgG (−) and LogMAR < 1		
		β	*p* value		β	*p* value
NFL	Baseline	1.6(1.6)	0.996	Δ2–Δ1	−2.9(2)	0.487
	2–5 months	−1.3(1.3)	0.975	Δ3–Δ2	−0.2(0.8)	1.000
	6–18 months	−1.4(1.5)	0.984	Δ3–Δ1	−3(2)	0.394
GCL	Baseline	0(2)	1.000	Δ2–Δ1	−2.7(1.6)	0.278
	2–5 months	−2.7(2.2)	0.66	Δ3–Δ2	−0.8(0.8)	0.987
	6–18 months	−3.5(2.4)	0.471	Δ3–Δ1	−3.5(2)	0.253
IPL	Baseline	0.6(1.5)	1.000	Δ2–Δ1	−2.2(1.2)	0.238
	2–5 months	−1.6(1.6)	0.93	Δ3–Δ2	−0.2(0.8)	1.000
	6–18 months	−1.8(1.6)	0.735	Δ3–Δ1	−2.4(1.4)	0.268
INL	Baseline	2.6(1.3)	0.142	Δ2–Δ1	−0.1(0.7)	1.000
	2–5 months	2.5(1.3)	0.165	Δ3–Δ2	0.6(0.4)	0.402
	6–18 months	3.1(1.4)	0.068	Δ3–Δ1	0.6(0.5)	0.762
OPL	Baseline	2.2(1)	0.097	Δ2–Δ1	−0.4(0.7)	1.000
	2–5 months	1.8(1.3)	0.446	Δ3–Δ2	0.3(0.7)	1.000
	6–18 months	2.1(1.2)	0.246	Δ3–Δ1	−0.1(0.8)	1.000
ONL	Baseline	1.1(3.3)	1.000	Δ2–Δ1	2.6(1.7)	0.375
	2–5 months	3.6(3.1)	0.716	Δ3–Δ2	−2.2(1.2)	0.198
	6–18 months	1.5(2.7)	1.000	Δ3–Δ1	0.4(1.4)	1.000
ORL	Baseline	1.5(1)	0.329	Δ2–Δ1	0.2(0.9)	1.000
	2–5 months	1.8(1)	0.244	Δ3–Δ2	−0.5(0.8)	1.000
	6–18 months	1.3(0.7)	0.238	Δ3–Δ1	−0.2(0.8)	1.000

Table S4. Changes of thickness in each retinal layer between group 2 and 4.

Retinal layer	Difference in thickness Between NMO-IgG (+) and LogMAR ≥ 1 vs. NMO-IgG (−) and LogMAR ≥ 1			Changes in thickness difference between NMO-IgG (+) and LogMAR ≥ 1 vs. NMO-IgG (−) and LogMAR ≥ 1		
		β	*p* value		β	*p* value
NFL	Baseline	−0.6(2.1)	1.000	Δ2–Δ1	−2.7(2.1)	0.627
	2–5 months	−3.2(2)	0.317	Δ3–Δ2	0.6(1.5)	1.000
	6–18 months	−2.7(1.7)	0.374	Δ3–Δ1	−2.1(2)	0.903
GCL	Baseline	0.6(3)	1.000	Δ2–Δ1	−5.1(2.6)	0.142
	2–5 months	−4.4(2.6)	0.281	Δ3–Δ2	−2(1.6)	0.618
	6–18 months	−6.4(2.8)	0.07	Δ3–Δ1	−7.1(3)	0.057

IPL	Baseline	−1.6(2.3)	1.000	Δ2–Δ1	−2.3(1.9)	0.63
	2–5 months	−0.8(1.9)	1.000	Δ3–Δ2	−1.3(1.2)	0.846
	6–18 months	−2(1.8)	0.775	Δ3–Δ1	−3.6(1.8)	0.139
INL	Baseline	0.5(1.1)	1.000	Δ2–Δ1	−0.5(1.3)	1.000
	2–5 months	0(1.5)	1.000	Δ3–Δ2	1.2(0.9)	0.552
	6–18 months	1.2(1.2)	0.948	Δ3–Δ1	0.7(1)	1.000
OPL	Baseline	−0.1(1.2)	1.000	Δ2–Δ1	−0.4(1)	1.000
	2–5 months	−0.6(1.4)	1.000	Δ3–Δ2	2.1(0.7)	**0.013**
	6–18 months	1.5(1.2)	0.58	Δ3–Δ1	1.6(0.9)	0.215
ONL	Baseline	−2.2(4.2)	1.000	Δ2–Δ1	2.9(2.2)	0.564
	2–5 months	0.7(4.2)	1.000	Δ3–Δ2	−2.5(1.2)	0.122
	6–18 months	−1.8(4.4)	1.000	Δ3–Δ1	0.4(1.8)	1.000
ORL	Baseline	0.3(0.9)	1.000	Δ2–Δ1	1(1.1)	1.000
	2–5 months	1.3(0.9)	0.45	Δ3–Δ2	−1.2(0.8)	0.432
	6–18 months	0.1(1.1)	1.000	Δ3–Δ1	−0.3(1.2)	1.000

Table S5. Changes of thickness in each retinal layer between group 1 and 4.

Retinal layer	Difference in thickness Between NMO-IgG (+) and LogMAR ≥ 1 vs. NMO-IgG (−) and LogMAR < 1			Changes in thickness difference between NMO-IgG (+) and LogMAR ≥ 1 vs. NMO-IgG (−) and LogMAR < 1		
		β	*p* value		β	*p* value
NFL	Baseline	−1.1(1.4)	1.000	Δ2–Δ1	−5.5(1.3)	**< 0.001**
	2–5 months	−6.6(1.2)	**< 0.001**	Δ3–Δ2	−0.5(1.4)	1.000
	6–18 months	−7(1.3)	**< 0.001**	Δ3–Δ1	−6(1.5)	**< 0.001**
GCL	Baseline	−1.4(2.4)	1.000	Δ2–Δ1	−9.4(2.3)	**< 0.001**
	2–5 months	−10.8(1.9)	**< 0.001**	Δ3–Δ2	−1.9(1.3)	0.45
	6–18 months	−12.7(2.2)	**< 0.001**	Δ3–Δ1	−11.3(2.8)	**< 0.001**
IPL	Baseline	−0.6(1.9)	1.000	Δ2–Δ1	−5.3(1.8)	**0.009**
	2–5 months	−5.9(1.7)	**0.002**	Δ3–Δ2	−1.8(1.1)	0.301
	6–18 months	−7.7(1.3)	**< 0.001**	Δ3–Δ1	−7.1(1.7)	**< 0.001**
INL	Baseline	0.7(0.9)	1.000	Δ2–Δ1	−0.2(1.2)	1.000
	2–5 months	0.5(1.5)	1.000	Δ3–Δ2	1.2(0.9)	0.464
	6–18 months	1.7(1.2)	0.506	Δ3–Δ1	1(0.8)	0.594
OPL	Baseline	2.2(0.9)	0.052	Δ2–Δ1	−1.5(0.8)	0.221
	2–5 months	0.8(0.9)	1.000	Δ3–Δ2	0.9(0.6)	0.396
	6–18 months	1.6(0.9)	0.196	Δ3–Δ1	−0.6(0.8)	1.000
ONL	Baseline	−2.5(3.1)	1.000	Δ2–Δ1	4.2(1.7)	**0.041**
	2–5 months	1.6(3.3)	1.000	Δ3–Δ2	−0.7(0.8)	1.000
	6–18 months	0.9(3.1)	1.000	Δ3–Δ1	3.5(1.6)	0.09
ORL	Baseline	−0.1(1)	1.000	Δ2–Δ1	1.8(1)	0.218
	2–5 months	1.7(0.7)	**0.036**	Δ3–Δ2	−1.1(0.7)	0.32
	6–18 months	0.6(0.9)	1.000	Δ3–Δ1	0.7(1.1)	1.000

Table S6. Changes of thickness in each retinal layer between group 2 and 3.

Retinal layer	Difference in thickness Between NMO-IgG (+) and LogMAR < 1 vs. NMO-IgG (−) and LogMAR ≥ 1			Changes in thickness difference Between NMO-IgG (+) and LogMAR < 1 vs. NMO-IgG (−) and LogMAR ≥ 1		
		β	*p* value		β	*p* value
NFL	Baseline	−2.1(2.3)	1.000	Δ2–Δ1	0(2.7)	1.000
	2–5 months	−2(2.2)	1.000	Δ3–Δ2	−0.9(0.9)	1.000

	6–18 months	−2.9(2)	0.447	Δ3–Δ1	0.8(2.4)	1.000
GCL	Baseline	−2.1(2.7)	1.000	Δ2–Δ1	1.6(1.9)	1.000
	2–5 months	−3.7(2.9)	0.618	Δ3–Δ2	0.9(1.2)	1.000
	6–18 months	−2.9(3.2)	1.000	Δ3–Δ1	0.8(2.4)	1.000
IPL	Baseline	−2.8(1.9)	0.406	Δ2–Δ1	0.8(1.3)	1.000
	2–5 months	−3.6(1.9)	0.162	Δ3–Δ2	−0.3(0.9)	1.000
	6–18 months	−3.8(2)	0.179	Δ3–Δ1	1.1(1.5)	1.000
INL	Baseline	−2.4(1.5)	0.302	Δ2–Δ1	−0.4(0.7)	1.000
	2–5 months	−2(1.4)	0.462	Δ3–Δ2	−0.6(0.5)	0.693
	6–18 months	−2.6(1.4)	0.177	Δ3–Δ1	0.2(0.8)	1.000
OPL	Baseline	0.1(1.4)	1.000	Δ2–Δ1	0.6(1)	1.000
	2–5 months	−0.5(1.7)	1.000	Δ3–Δ2	−1.5(0.8)	0.226
	6–18 months	−2(1.5)	0.513	Δ3–Δ1	2.1(0.9)	0.066
ONL	Baseline	−1.4(4.9)	1.000	Δ2–Δ1	1.3(2.2)	1.000
	2–5 months	−2.7(4.7)	1.000	Δ3–Δ2	4(1.5)	**0.023**
	6–18 months	1.3(4.6)	1.000	Δ3–Δ1	−2.7(1.6)	0.315
ORL	Baseline	−2(0.9)	0.105	Δ2–Δ1	−0.6(1)	1.000
	2–5 months	−1.4(1.2)	0.786	Δ3–Δ2	0.6(0.9)	1.000
	6–18 months	−0.8(1)	1.000	Δ3–Δ1	−1.2(1)	0.693

© 2020 by the authors. Licensee MDPI, Basel, Switzerland. This article is an open access article distributed under the terms and conditions of the Creative Commons Attribution (CC BY) license (http://creativecommons.org/licenses/by/4.0/).
